# What validation tests can be done by the clinical medical physicist while waiting for the standardization of quantitative SPECT/CT imaging?

**DOI:** 10.1186/s40658-022-00434-6

**Published:** 2022-02-05

**Authors:** Hanna Piwowarska-Bilska, Aleksandra Supińska, Bożena Birkenfeld

**Affiliations:** 1 Nuclear Medicine Department, Pomeranian Medical University, Szczecin, Poland; 2 Clinical Nuclear Medicine Department, University of Zielona Gora, Multi-Specialist Regional Hospital, Gorzow Wielkopolski, Poland

**Keywords:** Absolute quantification, SPECT/CT, Absolute SUV, QSPECT

## Abstract

**Objective:**

The aim of the study was to assess the accuracy of quantitative SPECT/CT imaging in a clinical setting and to compare test results from two nuclear medicine departments.

**Methods:**

Phantom studies were carried out with two gamma cameras manufactured by GE Healthcare: Discovery NM/CT 670 and NM/CT 850, used in two nuclear medicine departments. The data were collected using a cylindrical uniform phantom and a NEMA/IEC NU2 Body Phantom, filled with ^99m^Tc-pertechnetate.

**Results:**

The convergence of activity concentration recovery was validated for the two gamma cameras operating in two medical centers using the cylindrical uniform phantom. The comparison of results revealed a 5% difference in the background calibration factor Bg. cal; 6% difference in COV, and a 0.6% difference in total activity deviation ∆*A*_tot_. Recovery coefficients (RC_max_) for activity concentration in spheres of the NEMA/IEC NU2 Body Phantom were measured for different image reconstruction techniques. RC_max_ was in the range of 0.2–0.4 for the smallest sphere (ϕ 10 mm), and 1.3–1.4 for the largest sphere (ϕ 37 mm). Conversion factors for SUVmax and SUVmean for the gamma camera systems used were 0.99 and 1.13, respectively.

**Conclusions:**

(1) Measurements taken in our study confirmed the clinical suitability of 5 parameters of image quality (Bg. cal—background calibration factor, ∆*A*_tot_—total activity deviation, COV—coefficient of variation used for image noise assessment, *Q*_H_—hot contrast, AM—accuracy of measurements, or RC—recovery coefficient) for the validation of SPECT/CT system performance in terms of correct quantitative acquisitions of images. (2) This work shows that absolute SPECT/CT quantification is achievable in clinical nuclear medicine centers. Results variation of quantitative analyses between centers is mainly related to the use of different reconstruction methods. (3) It is necessary to standardize the technique of measuring the SUV conversion factor obtained with different SPECT/CT scanners.

## Background

The integrated diagnostic SPECT/CT (single-photon emission computed tomography/computed tomography) systems with iterative algorithms for image reconstruction allow for the clinical use of quantitative SPECT [1]. SPECT is a quantitative imaging technique and therefore requires a common quality control procedure to maintain the accuracy and precision of quantitation. The standard methodology for evaluating the performance of quantitative SPECT/CT systems has been established [2]. However, so far, no guidelines for quantitative SPECT/CT systems harmonization have been published. Many studies showed the need for harmonization of quantitative SPECT/CT scanners across centers [3–5]. There are differences in the calibration of these systems, in the reconstruction methods and in the correction techniques being applied. Software from different vendors may also produce different quantitative results from the same SPECT system.

Repeatability and reproducibility are essential requirements for any quantitative measurement. Repeatability in SPECT relates to the uncertainty in obtaining the same result in the same patient examined more than once on the same system. Reproducibility relates to the variation that results when different conditions are used to make the measurements, for example, different gamma camera systems. Standard uptake value (SUV) is a widely available and easy-to-use quantifier of radioactivity concentration in SPECT images. Physical and clinical conditions for its measurement still need to be standardized. A measured SUV cannot be used as an absolute number. SUVmax, which is preferred by physicians, depends, among other things, on the chosen SPECT/CT image reconstruction technique.

Harmonization and standardization of quantitative test results have been successfully implemented for PET/CT (positron emission tomography/computed tomography) imaging technique. From the beginning of its existence, this diagnostic method was considered a quantitative one and, therefore, absolute quantification was the overriding goal of the development of PET technology. However, variability in methodology across centers prohibited the exchange of SUV data. In 2006, the EANM (European Association of Nuclear Medicine) started the EARL (EANM Research Ltd. Program), i.e., the multicenter program of standardization and harmonization and accreditation of PET/CT scanners. The EANM/EARL accreditation program has been developed to facilitate comparisons of quantitative PET parameters in multicenter studies or at medical centers equipped with several PET systems [6]. Standardization includes the unification procedures of patient preparation, scan acquisition, image reconstruction and data analysis settings. The harmonizing standards are based on the precise calibration of PET scanners. To obtain and maintain the EARL accredited status, PET centers are required to complete and submit two phantoms scans for calibration quality control (using a uniform cylindrical phantom), and image quality control (using a NEMA NU2-2007 Body Phantom) [7]. The standardization of quantitative PET results is also done by committees and the working group established by the Society of Nuclear Medicine and Molecular Imaging and the Radiological Society of North America. The results of the integrated works of international teams of experts are the guidelines for performing, interpreting and reporting the results of PET/CT studies [8, 9]. Selected and proven procedures, quality control tests and tools used to harmonize PET scanners could be used for QSPECT harmonization.

The tests presented in the paper were performed by the authors using ^99m^Tc (technetium-99m), the most widely used radioisotope for diagnostic studies in nuclear medicine. ^99m^Tc sources are used for most of the quality control tests of gamma camera detectors recommended by the manufacturers.

Accuracy calibration and harmonization of SPECT/CT systems should improve the quality of dosimetric measurements performed on patients' images to estimate the doses absorbed by individual organs. Radiation dose optimization in diagnostic imaging and the need for individual planning of radioisotope therapies, recommended by the European Directive 2013/59/Euratom [10], lead to the rising importance of clinical internal dosimetry in nuclear medicine departments.

Selected issues related to the quantitative analysis of SPECT/CT images were addressed in the present study: (1) What tests can be done to assess the performance of a SPECT/CT gamma camera used for quantitative imaging? (2) Is the level of accuracy of activity measurement obtained by manufacturers in their research laboratories achievable in the clinical facilities of nuclear medicine? (3) How can the repeatability of SUV measurements for two different SPECT/CT systems be tested?

## Methods

The present study was conducted in two departments of nuclear medicine: the Clinical Nuclear Medicine Department of the University of Zielona Gora, Multi-Specialist Regional Hospital in Gorzow Wielkopolski (Department 1), and in the Nuclear Medicine Department of the Pomeranian Medical University in Szczecin (Department 2). The data were collected using the cylindrical uniform phantom and the NEMA/IEC NU2 Body Phantom, filled with ^99m^Tc-pertechnetate. Phantom tests were performed using Discovery NM/CT 670 gamma camera operating in Department 1 and NM/CT 850 gamma camera operating in Department 2. Acquired data were processed on Xeleris 2 Workstation in Department 1 and on Xeleris 4 Workstation in Department 2. Activity measurements were performed with two different activity meters: Capintec CRC-55tR No. 158952 at Department 1 and Capintec CRC-55tR No. 550547 at Department 2. Two different operators (the physicists experienced in nuclear medicine) performed the phantom preparation, imaging and data analysis procedures, separately in each department.

**Cross-measurements**

- Cross-calibration of activity meter to SPECT/CT system
  Gamma camera sensitivities, checked regularly according to vendor recommendations in both departments, were measured using the GE Healthcare application: “Camera Sensitivity guided workflow test.” The camera sensitivity was measured using a Petri dish that was filled evenly (2–3 mm deep) with a homogeneous solution of ^99m^Tc-pertechnetate (90 MBq) in water.
- Comparison of the activity meters
  Both activity meters Capintec-55tR No. 158952 and No. 550547 had valid calibration certificates issued by The Laboratory of Radioactivity Standards of the Radioisotope Center POLATOM (Accredited Laboratory No. AP 120). The calibration certificates contained information on the results of the linearity test performed using ^99m^Tc sources. The deviation from linearity did not exceed 1.0% for the Capintec CRC-55tR No. 158952, and 0.5% for the Capintec CRC-55tR No. 550547, within the activity ranges tested by the accredited laboratory.
  In the study, the readings of both activity meters were checked using a calibration source prepared at Department 1 in Gorzów. One of the NEMA/IEC Body Phantom spherical inserts (with a diameter of 22 mm) was used as the calibration source. The calibration source was filled with ^99m^Tc-pertechnetate and measured with Capintec CRC-55tR No. 158952 activity meter at Department 1. Then, the same calibration source was measured with Capintec CRC-55tR No. 550547, after its delivery to Department 2 in Szczecin.

**1. Accuracy of measurement for activity in the cylindrical uniform phantom.**

**Fig. 1:**
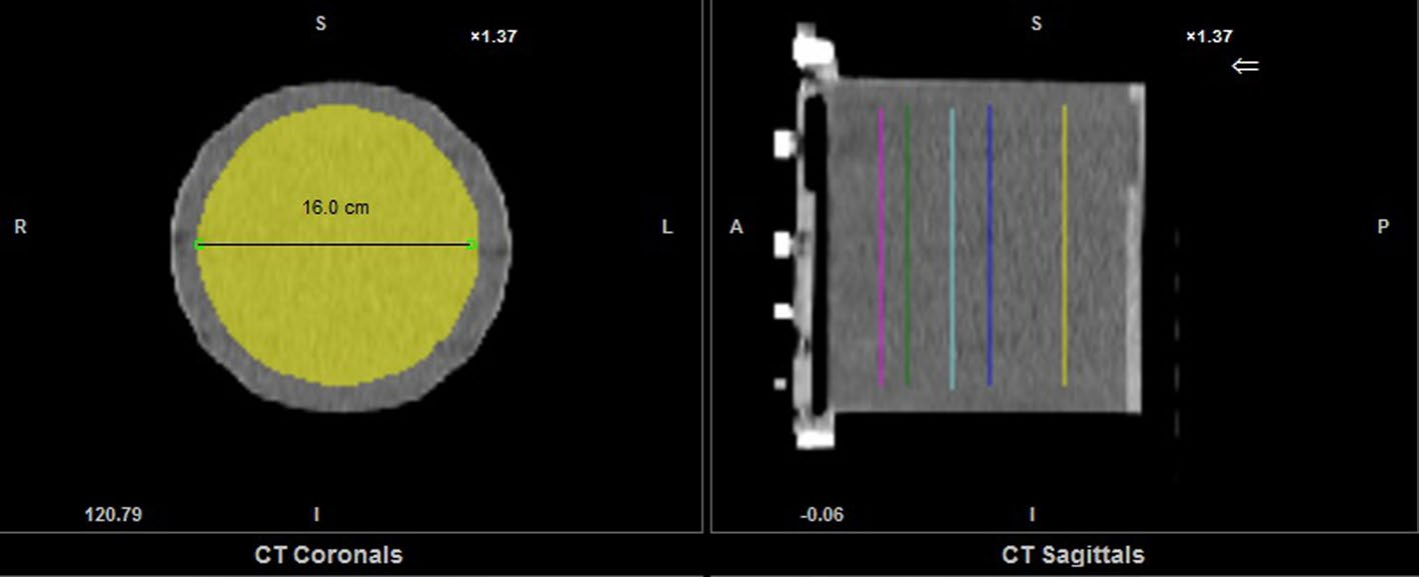
Image of the uniform cylindrical phantom with 5 circular regions of interest of 16 cm in diameter located at different levels along the long axis of the phantom, reconstructed with Q.Metrix application of Xeleris 2 software from GE Healthcare

The cylindrical uniform phantom, 18 cm high, 20 cm in diameter and 5640 ml volume, was filled with a homogeneous solution of the radioisotope (^99m^Tc) with activity of 294 MBq (Department 1) and 295 MBq (Department 2) at the moment of scan start. The same SPECT/CT acquisitions of the phantom were performed in the two medical departments. The acquisition parameters were as follows: low-energy high-resolution collimator; the photopeak emission energy window: 140.5 keV ± 10%; and the scatter window: 120 ± 5% keV. SPECT images were acquired with 60 projections over 360°, 180° per detector, step of 6°, 20 s/projection. Acquired images were reconstructed using 24 iterations, 4 subsets, a Gaussian smoothing with a full width at half maximum (FWHM) of 7.5 mm, and corrections: AC (attenuation correction), SC (scatter correction), RR (resolution recovery). The following parameters were established for the cylindrical uniform phantom, as proposed by Gnesin et al. [11]:

- The SPECT-to-local activity meter cross-calibration (Bg. cal) was tested by calculating ratio of the activity concentration measured in the reconstructed SPECT phantom background (*a*_*c*,bg_ [MBq/ml]) to the expected activity concentration of liquid filling the phantom (*A*_*c*,bg_ [MBq/ml]), measured during phantom preparation.
  1 $${\text{Bg}}.\,{\text{cal}} = \frac{{a_{c,bg} }}{{A_{c,bg} }}$$
  *a*_*c*,bg_ was evaluated as the mean value for 5 circular regions of interest (ROIs) of 16 cm in diameter centered on the cylinder axis of the phantom placed at different axial locations (Fig. 1).
- The image noise was evaluated using the coefficient of variation (COV), which was the ratio of standard deviation (*σ*_bg_) to the average signal measured in the phantom background (*a*_*c*,*bg*_).
  2 $${\text{COV}}\left( \% \right) = \frac{{\sigma_{bg } }}{{a_{c, bg} }} \times 100 ,$$
  Standard deviation (*σ*_*bg*_) was calculated according to the equation:
  3 $$\sigma_{bg } = \sqrt {\mathop \sum \limits_{n = 1}^{n} \frac{{a_{c, bg,n} - a_{c, bg} }}{n - 1}} , \quad n = 5,$$
  where $a_{c, bg,n}$ is mean activity concentration in the n circular ROI calculated with Q.Metrix program.
- The total activity deviation $\left( {\Delta A_{{{\text{tot}}}} \left( \% \right)} \right)$ was calculated:
  4 $$\Delta A_{{{\text{tot}}}} \left( \% \right) = \frac{{A_{{\text{tot,rec}}} - A_{{{\text{tot}}}} }}{{A_{{{\text{tot}}}} }} \times 100$$
  where *A*_tot,rec_ [MBq] is the total recovered activity in the phantom measured in the image with Q.Metrix application of Xeleris from GE Healthcare. During the measurement of *A*_tot,rec_, the maximum rectangular VOI covering the entire area of the phantom was drawn on the middle coronal slice of the reconstructed image. $A_{{{\text{tot}}}}$ [MBq] is the total activity in the phantom measured with an activity meter during phantom preparation.

**2. Accuracy of measurements for the activity in hot spherical inserts in the NEMA/IEC NU2 Body Phantom.**

In Department 1 and then in Department 2, identical SPECT/CT acquisitions were performed for the NEMA IEC Body Phantom prepared consistently with instructions on laboratory measurements provided in the white paper published by GE Healthcare [12]. The following acquisition parameters were used: low-energy high-resolution collimator; the photopeak emission energy window: 140.5 keV ± 10%; and the scatter window: 120 ± 5% keV. SPECT images were acquired with 120 projections over 360°, 180° per detector, step of 3°, 30 s/projection. The NEMA IEC Body Phantom, loaded with a ^99m^Tc solution, was used to assess quantification accuracy using 6 different sphere sizes. Six spherical inserts (10, 13, 17, 22, 28 and 37 mm in diameter) were filled with an activity concentration 8 times higher than the activity concentration present in the phantom cylinder. The total phantom activity at the time of the scan was 386.8 MBq in Department 1, and 386.6 MBq in Department 2.

**(A)**

Acquired images were reconstructed using 6 subsequent iterative techniques:

- 2 iterations, 10 subsets; AC, SC, RR corrections; no filtering,
- 2 iterations, 10 subsets; AC, SC, RR corrections; Butterworth filter (cutoff frequency of 0.48 cycles/cm and an order of 10),
- 4 iterations, 10 subsets; AC, SC, RR corrections; no filtering,
- 4 iterations, 10 subsets; AC correction; no filtering.
- 5 iterations, 15 subsets; AC, SC, RR corrections; no filtering,
- 24 iterations, 8 subsets; AC, SC, RR corrections; no filtering.

**Fig. 2:**
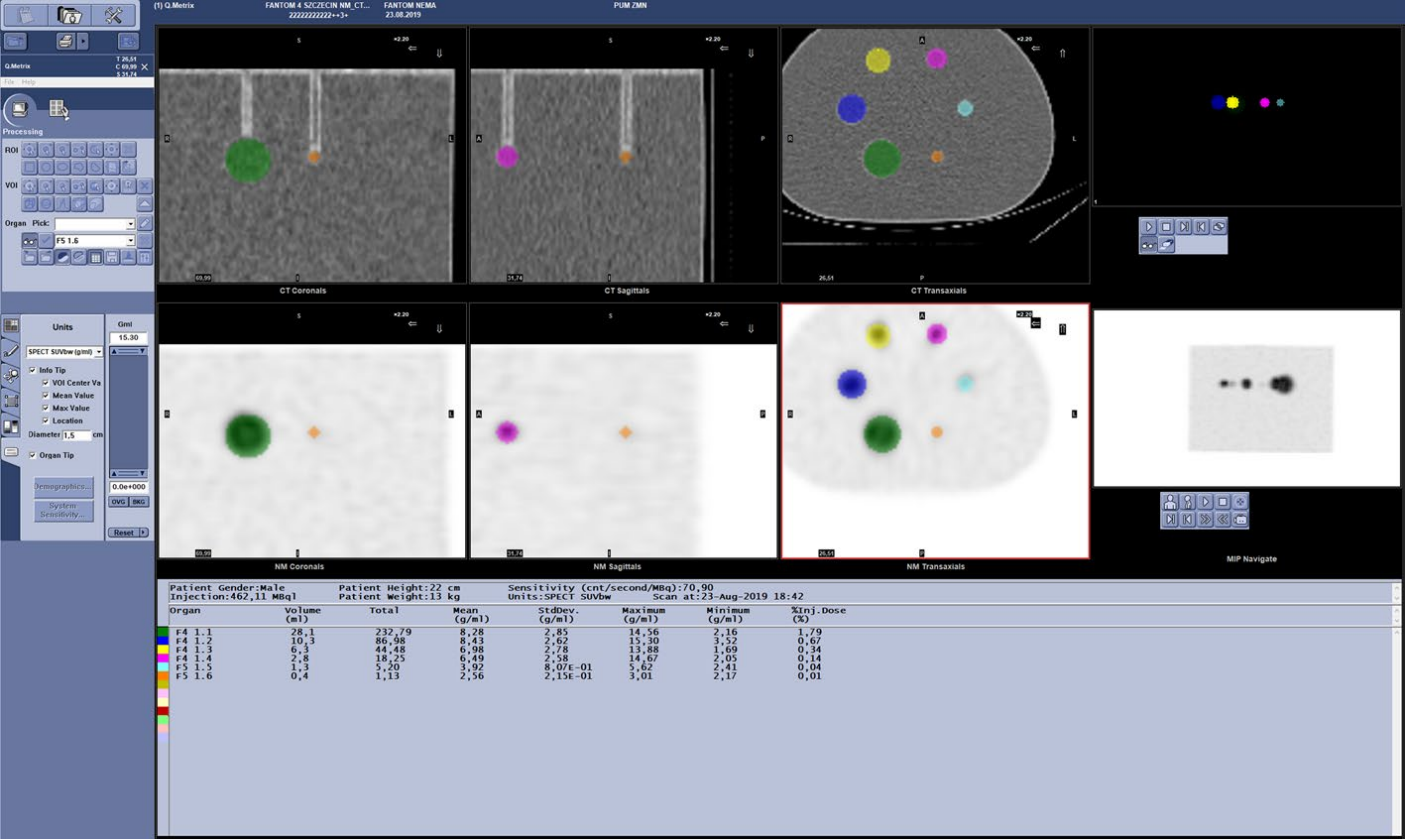
A screenshot of the Q.Metrix software during the measurement of hot spheres activity in the cross sections of the NEMA/IEC Body Phantom. Color rings correspond to VOI (volume of interest) for 6 hot spheres

Accuracy of measurements (AM) for activity of 6 spheres in the images of the phantom was evaluated from the formula [12]:

5 $${\text{AM}} = \left( {1 - \frac{{\left| {{\text{Loaded}}\,{\text{activity}} - {\text{Measured}}\,{\text{activity}}} \right|}}{{{\text{Loaded }}\,{\text{activity}}}}} \right) \times 100\%$$

where ${\text{Loaded}}\,{\text{activity}}$ [MBq]—the real activity measured with an activity meter at the time of acquisition, and ${\text{Measured }}\,{\text{activity}}$ [MBq]—the activity measured in SPECT/CT images with Q.Metrix from GE Healthcare for individual spherical inserts. Figure 2 presents the technique of radioactivity measurement for 6 hot spheres of the phantom during the analysis of reconstructed images.

**(B)**

Cross sections of the NEMA/IEC Body Phantom were additionally reconstructed using 7 subsequent techniques, with 4, 8, 12, 16, 20, 24, 48 iterations; 4 subsets; AC, SC, RR corrections; and no filtering. Recovery coefficient (RC) was evaluated for all 6 spheres and images acquired with 7 different reconstruction techniques [11]:

6 $${\text{RC}}_{j,\max } = \frac{{a_{c,sph,j,\max } }}{{A_{c,sph} }}$$

where *j* is the number of sphere, $a_{c,sph,j,\max } \left[ {\text{MBq/ml}} \right]$ is maximum activity concentration in the sphere measured with Q.Metrix, and $A_{c,sph}$ $\left[ {\text{MBq/ml}} \right]$ is activity concentration in the sphere established based on measurements taken with an activity meter.

A relative deviation between measured and calculated sphere’s activity value was defined by:

7 $${\text{BIAS}}_{j} = \left| {{\text{RC}}_{j,\max } - 1} \right| \times 100\%$$

The average BIAS was calculated as the arithmetic mean of all BIASj for 6 spheres.

Another notation for the BIAS definition (expressed using the quantity “Accuracy of measurements—AM”) was the following:

8 $${\text{BIAS}} = 100\% \, - {\text{ AM}}$$

For the NEMA/IEC Body Phantom, the image noise, evaluated using the coefficient of variation (COV), was assessed with Q.Volumetrix, another Xeleris software application. The formula for calculating COV was analogous to formula (2), except that in this case, the average activity concentration was obtained by averaging the signal from the six cubic regions of interest (side of 40 mm) placed in the uniform background surrounding the six spheres. The value of *σ*_*bg*_ in this method was calculated as the standard deviation of the mean of the results of the activity concentration measurement for all the six cubic background VOIs.

The following parameters were also evaluated for the images of the NEMA/IEC Body Phantom (reconstructed using 16 iterations 4 subsets; AC, SC, RR corrections; no filtering):

- activity concentration in the background,
- activity concentration in the 22-mm-diameter hot sphere,
- hot contrast (*Q*_*H*,22_) for the 22-mm-diameter sphere, from the formula [11]:

9 $$Q_{H,j} \left( \% \right) = \frac{{\left( {a_{c,sph,j} /a_{c,bg} } \right) - 1}}{{\left( {A_{c,sph } /A_{c,bg} } \right) - 1}} \times 100$$

where $a_{c,bg}$ is activity concentration in the background measured with Q.Metrix in the image of the phantom, and $A_{c,bg}$ is activity concentration of the radioactive liquid in which hot spheres are immersed (background) measured with an activity meter.

**3. Comparison of SUVs acquired for a single calibration source.**

Measurements of the sensitivity of the gamma cameras, according to the manufacturer's recommendations, in both departments were performed with locally measured sources. The SUV was calculated for the same source for two purposes: to compare the cameras’ sensitivities and to verify the correctness of calculations of Q.Metrix applications in both systems. Verifying the correctness of the calculation of clinical parameters (like SUV) can be helpful when testing the software of newly purchased systems.

To determine the conversion factor for SUVmax and SUVmean registered in studies with different gamma cameras, two identical SPECT/CT acquisitions and reconstructions of the same ^99m^Tc calibration source were performed in two nuclear medicine departments. The calibration source was a 22-mm-diameter hot spherical insert of the NEMA/IEC Body Phantom with activity of 20.03 MBq prepared and measured with Capintec CRC-55tR (No. 158952) activity meter at Department 1.

**Fig. 3:**
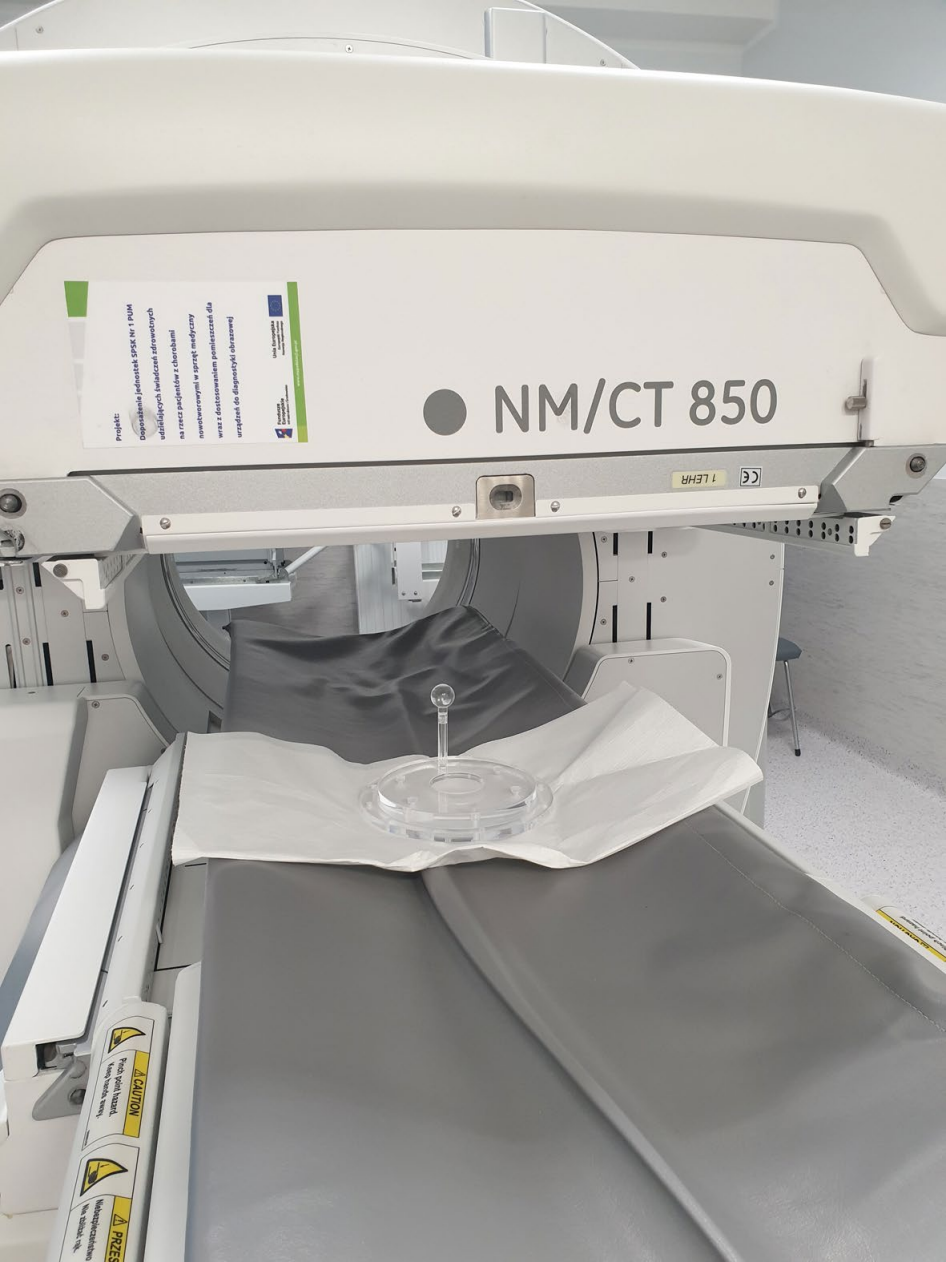
Acquisition of the calibration source in Department 2

The acquisition of the calibration source was carried out with Discovery NM/CT 670 gamma camera in Department 1, and a few hours later with NM/CT 850 gamma camera in Department 2 (Fig. 3). The images acquired with different gamma cameras were reconstructed using the same method: 4 iterations, 10 subsets; AC, SC, RR corrections; no filtering.

## Results

**Cross-measurements**

- Cross-calibration of activity meter to SPECT/CT system
  The sensitivities of the gamma cameras, measured before the quantitative acquisitions, were 72.2 cps/MBq for Discovery NM/CT 670 gamma camera and 70.9 cps/MBq for NM/CT 850 gamma camera.
- Comparison of the activity meters
  The activity of radiopharmaceutical inside the 22-mm sphere was measured in Department 1 with a Capintec CRC-55tR meter No. 158952, and it was 20.03 MBq. The activity of this sphere measured in Department 2 with a Capintec CRC-55tR No. 550547 was 8.20 MBq. After taking into account a decay correction of the radionuclide, the measurement results of the same source for the two meters were: 20.03 MBq for the Capintec CRC-55tR No. 158952 and 21.01 MBq for the Capintec CRC-55tR No. 550547 m. The percentage difference in the readings was therefore 4.7%, and the ratio of Department 2 meter reading to Department 1 meter reading was 1.05.

**1. Accuracy of measurement for activity in the cylindrical uniform phantom.**

Table 1 presents data on the accuracy of activity concentration recovery in the images of the cylindrical uniform phantom acquired with two gamma cameras in two nuclear medicine departments. The comparison of results for the two gamma cameras revealed a 5% difference in the background calibration factor Bg. cal; 6% difference in COV; and a 0.6% difference in total activity deviation ∆*A*_tot_. Activity concentration measured for the ^99m^Tc solution with an activity meter and then measured in the image with Q.Metrix was identical for both gamma cameras.

**Table 1:** Background calibration factor (Bg. cal), coefficient of variation (COV), total activity deviation (Δ*A*_tot_), background activity concentration (*A*_*c,bg*_) measured with the activity meter and background activity concentration (*a*_*c,bg*_)—average of the 5 ROIs measured in the image with Q.Metrix, for two SPECT/CT acquisitions of the cylindrical uniform phantom in two nuclear medicine departments

Gamma camera, medical center	Coefficient				
	Background calibration factor Bg. cal	Coefficient of variation COV (%)	Total activity deviation Δ*A*_tot_ (%)	Activity concentration in the background *A*_*c,bg*_ (MBq/ml) (measured with an activity meter)	Activity concentration in the background *a*_*c,bg*_ (MBq/ml) (measured in the image with Q.Metrix, average of the 5 ROIs)
Discovery 670 Department 1	1.192	4	− 1.02	0.052	0.062
NM/CT 850 Department 2	1.135	10	− 0.45	0.052	0.059

**2. Accuracy of measurements for the activity in hot spherical inserts in the NEMA/IEC NU2 Body Phantom.**

**(A)**

**Fig. 4:**
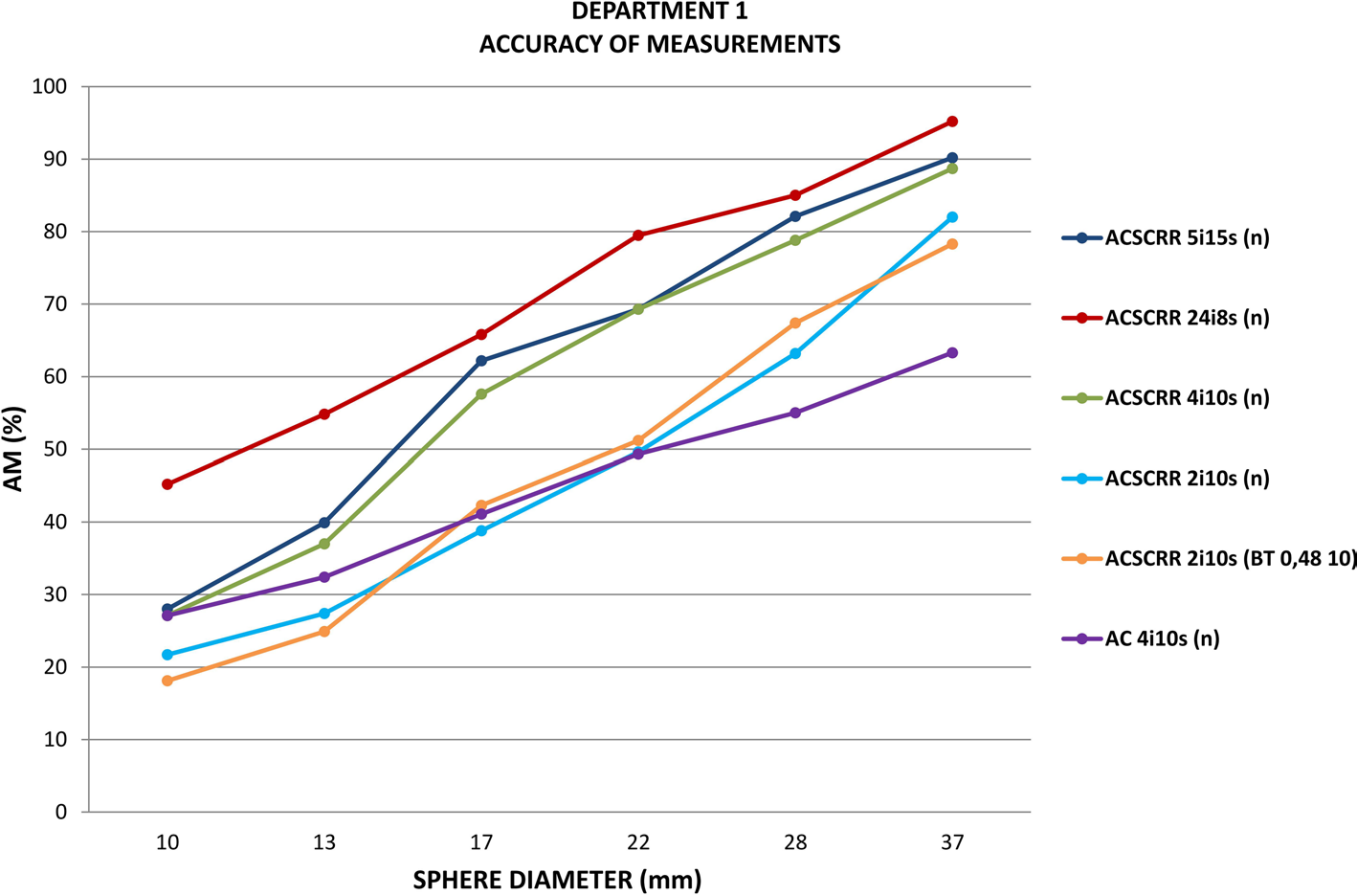
Accuracy of measurements (AM) for activity in 6 spherical inserts of the NEMA/IEC Body Phantom scanned with Discovery NM/CT 670 gamma camera in Department 1

Figure 4 presents the accuracy of measurements for activity concentration in 6 hot spherical inserts of the NEMA/IEC Body Phantom recorded with Discovery NM/CT 670 gamma camera in Department 1. The highest accuracy of measurement for activity concentration was obtained when using an image reconstruction technique with 24 iterations and 8 subsets, corrections: AC, SC and RR. Another two reconstruction techniques offering the relatively high accuracy of measuring the activity concentration in spheres involved 5 iterations with 15 subsets or 4 iterations with 10 subsets. The use of reconstruction techniques with fewer than 4 iterations was associated with a significant reduction in the accuracy of measurement for the activity in all 6 spheres. The use of the reconstruction technique with just 2 iterations reduced the accuracy of measurement for the largest sphere (ϕ = 37 mm) by 8%. When the Butterworth filter (0.48 10) was used in the technique with 2 iterations, the accuracy of activity measurement dropped by another 4% for this hot sphere.

**Fig. 5:**
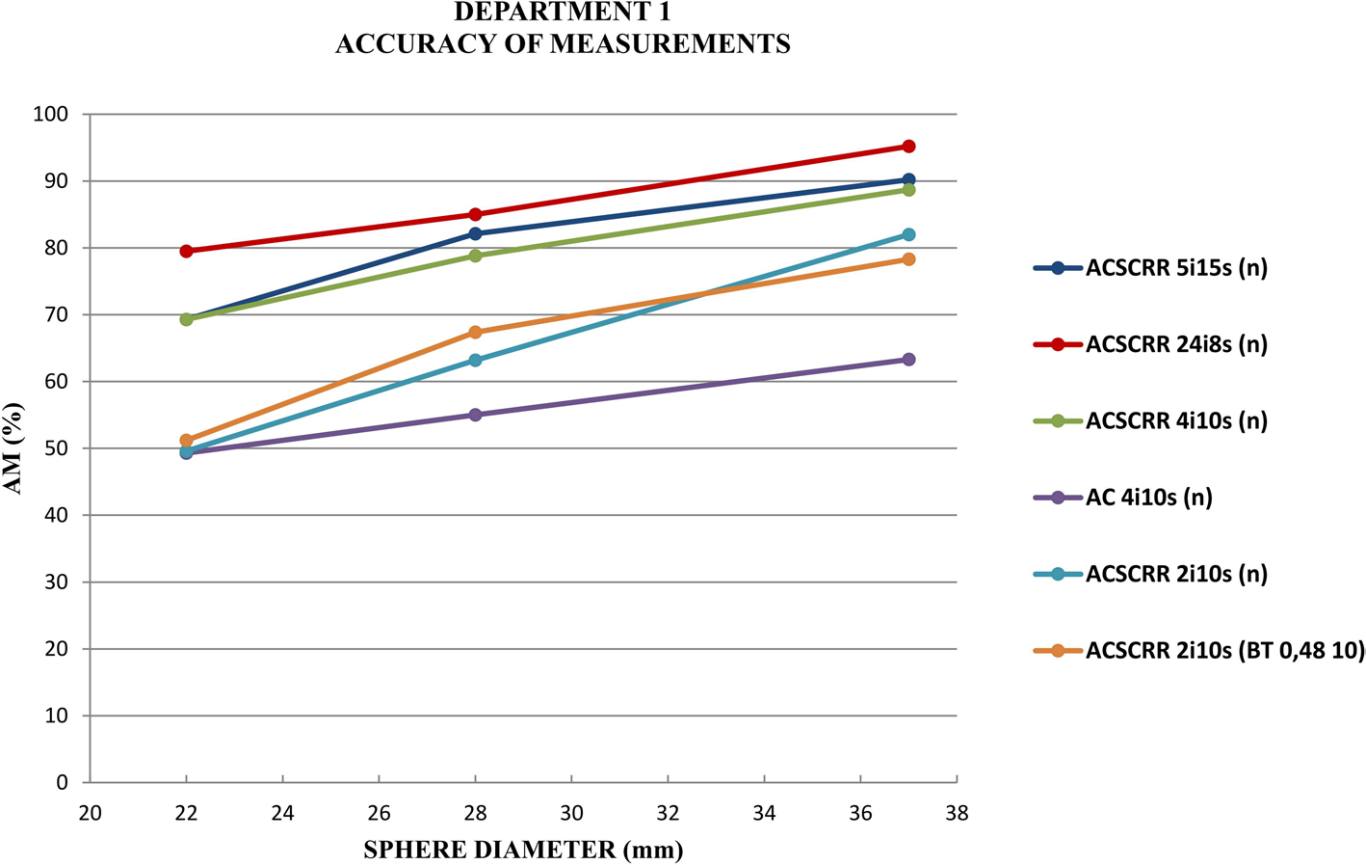
Accuracy of measurements for activity in the 3 largest spherical inserts of the NEMA/IEC Body Phantom scanned with Discovery NM/CT 670 gamma camera in Department 1

Figure 5 shows a fragment of the graph in Fig. 4. This figure was included in the paper to allow the comparison of similar findings in the literature [12]***.***

**Fig. 6:**
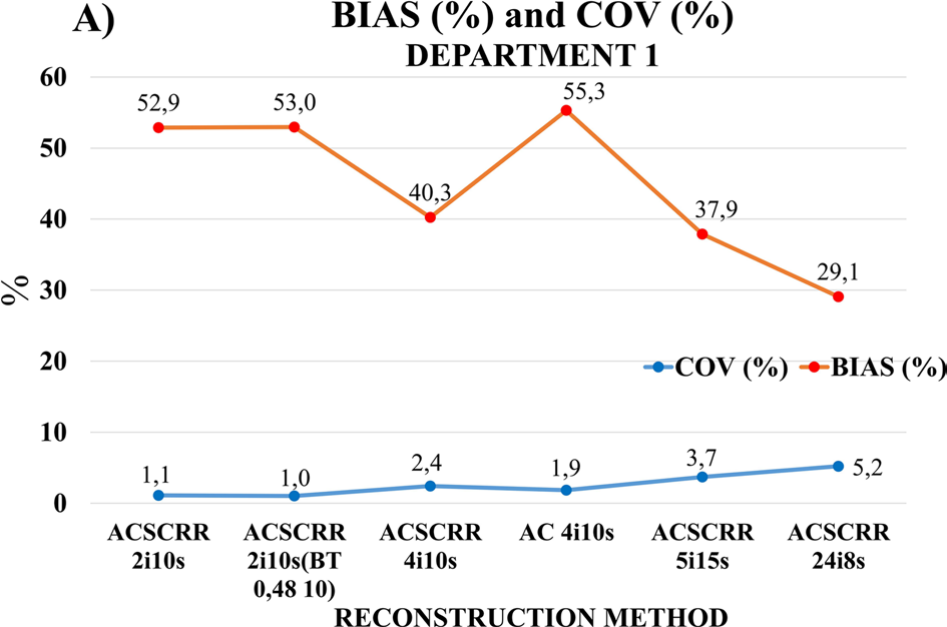
BIAS and COV plot showing average BIAS values of spheres activity concentration and COV average values for background, in the NEMA/IEC NU2 Body Phantom cross sections reconstructed with 6 methods compliant with the White Paper GE Healthcare [12]. The calculations were made with the Q.Metrix program for images acquired with Discovery NM/CT 670 at Department 1

Figure 6 shows the average values of deviations of the activity concentration from the expected value (BIAS) for all spheres and the average values of background noise (expressed by the COV coefficient) on the images of the NEMA/IEC NU2 phantom recorded in Department 1 and reconstructed using 6 different methods described in point 2.A) of Methods section. The maximum BIAS (55.3%) occurred for the 4 iterations 10 subsets method, without SC and RR corrections. The lowest BIAS (29.1%) was determined for the 24 iterations 8 subsets reconstruction method. The value of noise in the images increased (ranging from 1.1 to 5.2%) with the number of iterations. A slight decrease in noise was recorded for the 2 iterations 10 subsets method with the Butterworth reconstruction filter, as well as for the 4 iterations 10 subsets method without SC and RR corrections. The introduction of the filter to the reconstruction method with 2 iterations slightly reduced the noise level in the images (by 0.1% on average) with simultaneous increase in the averaged BIAS.

**(B)**

**Fig. 7:**
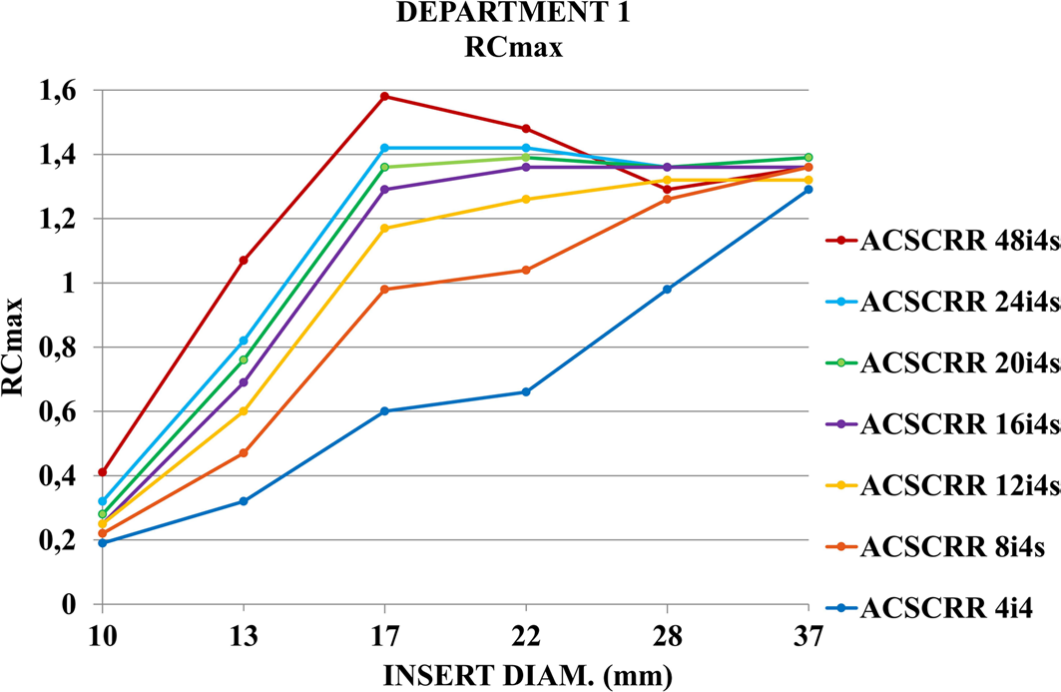
Recovery coefficients (RC_max_) for activity concentration in 6 spherical inserts of the NEMA/IEC NU2 Body Phantom scanned with Discovery NM/CT 670 gamma camera

Figure 7 presents recovery coefficients (RC_max_) for activity concentration in spheres of the NEMA/IEC NU2 Body Phantom. Images were acquired with Discovery NM/CT 670 gamma camera from GE Healthcare and reconstructed with 7 techniques described in a paper by Gnesin et al. [11]. The type of a reconstruction technique had a minor effect on the accuracy of measurement for the smallest (ϕ 10 mm) and largest (ϕ 37 mm) spheres. RC_max_ was in the range of 0.2–0.4 for the smallest sphere, and 1.3–1.4 for the largest sphere. The widest range of RC_max_ (0.6–1.6) was found for sphere no. 3 (ϕ 17 mm).

**Fig. 8:**
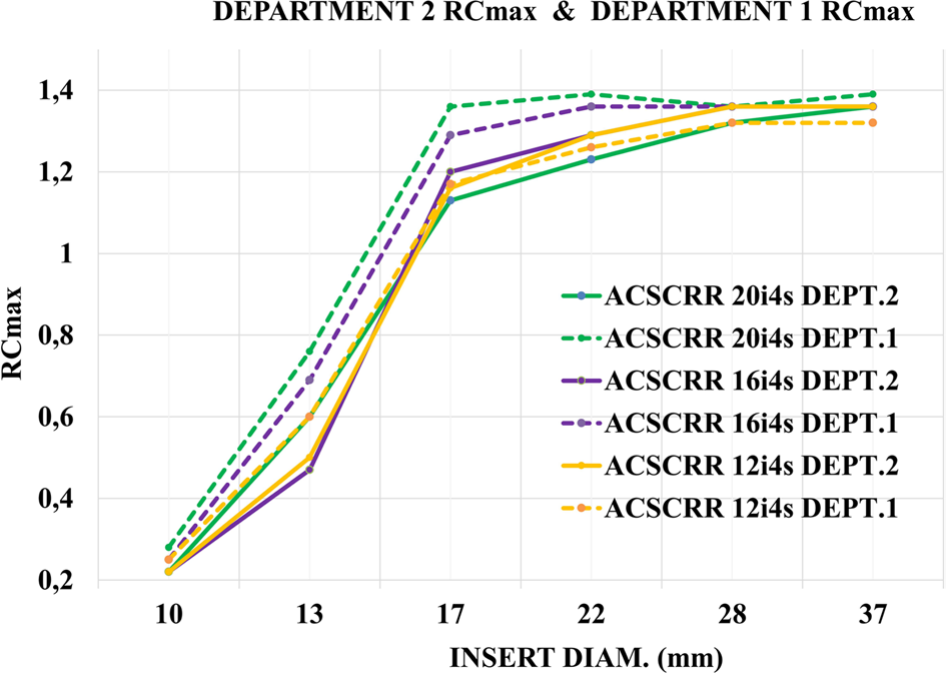
RC_max_ values as a function of the sphere diameter of the NEMA/IEC NU2 Body Phantom, for two gamma cameras in two departments. The dashed lines represent the results obtained by the gamma camera Discovery NM/CT 670 in Department 1; the continuous lines represent the results obtained by the gamma camera NM/CT 850 in Department 2

Figure 8 shows two sets of RC_max_ curves calculated for 6 hot spheres, for the NEMA/IEC NU2 phantom, obtained using two different gamma cameras (manufacturer GE Healthcare) at two departments. The images were reconstructed by 3 different methods using 12, 16 and 20 iterations. The absolute value of the difference between the RC_max_ values for the corresponding spheres and the reconstruction methods, obtained in these two departments, amounted to an average of 0.07, minimum 0, maximum 0.23. The highest RC_max_ difference concerned the sphere ϕ = 17 mm and the 20 iteration method. The absolute value of the difference between the RCmaxj values for the reconstruction methods with 12 and 20 iterations (averaged of 6 spheres) was 0.10 and 0.04, respectively, for Department 1 and Department 2. The beam of RC_max_ curves from Department 1 was broader than the beam from Department 2.

**Fig. 9:**
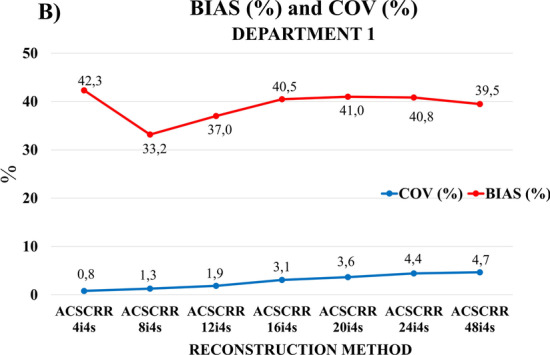
BIAS and COV plot showing average BIAS values of spheres activity concentration and COV average values for background, in the NEMA/IEC NU2 Body Phantom cross sections reconstructed with 7 methods compliant with the paper by Gnesin et al. [11]. The calculations were made with the Q.Metrix program for images acquired with Discovery NM/CT 670 at Department 1

Figure 9 shows the average values of deviations of the activity concentration from the expected value (BIAS) for all spheres and the average values of background noise (expressed by the COV coefficient) on the images of the NEMA/IEC NU2 phantom recorded in Department 1 and reconstructed using the 7 different methods described in point 2.B) of Methods section. The maximum BIAS (42.3%) occurred for the 4 iterations 4 subsets method. The lowest BIAS (33.2%) was determined for the 8 iterations 4 subsets reconstruction method. The amount of noise in the images grew steadily with the number of iterations. The range of COV values was: 0.8–4.7%.

Table 2 presents the calculated parameters of image quality obtained for the background and the 22-mm-diameter spherical insert of the NEMA/IEC NU2 Body Phantom measured with two gamma cameras, Discovery NM/CT 670 and NM/CT 850. Activity concentration measured for the ^99m^Tc solution in the 22-mm sphere with activity meters was identical for both phantoms. Activity concentrations measured in images of 22-mm sphere with Q.Metrix differed by 11% for two gamma cameras. Activity concentrations measured in the background of the phantom in the two nuclear medicine departments were very similar. Hot contrasts (*Q*_*H*,22_) measured in spherical insert were comparable (71 vs 76%).

**Table 2:** Parameters of image quality obtained for the 22-mm-diameter sphere and the background of the NEMA/IEC NU2 Body Phantom with two gamma cameras, Discovery NM/CT 670 and NM/CT 850

	Parameter				
	Activity concentration in the hot sphere *A*_*c*,sphere_ (22 mm) [MBq/ml] (measured with a meter)	Activity concentration in the hot sphere measured with Q.Metrix *a*_*c*,sphere_ (22 mm) [MBq/ml] (measured with Q.Metrix)	Hot contrast Q_H_ (%) 22-mm sphere (measured in the image)	Activity concentration in the background *A*_*c*,*bg*_ [MBq/ml] (measured with a meter)	Activity concentration in the background *a*_*c*,*bg*_ [MBq/ml] (measured with Q.Metrix, mean for 5 ROIs)
Discovery NM/CT 670 Department 1	0.32	0.42	71	0.04	0.07
NM/CT 850 Department 2	0.32	0.38	76	0.04	0.06

**Comparison of SUVs acquired for a single calibration source.**

Table 3 presents the comparison of calculated SUVmax and SUVmean for a calibration source—a hot sphere 22 mm in diameter (an insert of the NEMA/IEC Body Phantom) registered with two different GE Healthcare gamma cameras in the two nuclear medicine departments. The activity of the calibration source at the time of acquisition start was 13.9 MBq in Department 1 and 7.5 MBq in Department 2.

**Table 3:** SUVmax and SUVmean for ϕ 22-mm sphere obtained using two gamma cameras: Discovery NM/CT 670 and NM/CT 850, in the two medical departments. The radioactive decay corrections were included in the calculations

Gamma camera	Sphere volume Q.Metrix [ml]	Sphere activity Q.Metrix [MBq]	SUV_mean_ [g/ml]	SUV_max_ [g/ml]	SUV_min_ [g/ml]
Discovery NM/CT 670 Department 1	5.8	8.17	52.40	113.30	7.18
NM/CT 850 Department 2	5.8	6.47	59.05	112.62	6.56

Conversion factors for SUVmax and SUVmean were calculated for the gamma camera systems used:

10 $${\text{Conversion}}\,{\text{factor}}\,{\text{for}}\,{\text{SUVmax}}:\,\frac{{{\mathbf{SUVmax}}{ }\left( {{\text{NM}}/{\text{CT }}850{ }\,{\text{DEPARTMENT }}2} \right)}}{{{\mathbf{SUVmax}}{ }\left( {{\text{DISCOVERY}}\,{\text{NM}}/{\text{CT }}670\,{\text{DEPARTMENT }}1} \right)}} = 0.99$$

11 $${\text{Conversion factor for SUVmean:}}\,\frac{{{\mathbf{SUVmean}}{ }\left( {{\text{NM}}/{\text{CT }}850{\text{ DEPARTMENT }}2} \right)}}{{{\mathbf{SUVmean}}{ }\left( {{\text{DISCOVERY NM}}/{\text{CT }}670{\text{ DEPARTMENT }}1} \right)}} = 1.13$$

## Discussion

**1. Accuracy of measurement for activity in the cylindrical uniform phantom.**

The background calibration factors (Bg. cal) for the two gamma cameras, Discovery NM/CT 670 and NM/CT 850, were not within 10% of the expected value (1), but were convergent, and only a 5% difference was found between them. Gnesin et al. [11] reported that the background calibration factor (Bg. cal) was within 10% of the expected value, but the analysis was based on the images acquired during a two-times longer acquisition of the cylindrical uniform phantom. The increase in deviations from Bg. cal = 1 at low count density was observed in PET studies [13, 14]. Apart from the low count statistics, another reason for obtaining too large deviation of Bg. cal from the value of 1 could be limited (to two decimal places), the accuracy of the measurement of activity concentration obtained using the Q.Metrix application. Newer versions of GE Healthcare's Xeleris software already have a superior quantitative application: Q.Volumetrix.

The relatively low values of the coefficient of variation (COV%) for the background prove the high degree of homogeneity of the solution filling the phantom, and thus the correct preparation of uniform phantoms in Department 1 and Department 2. Total activity deviation (ΔAtot (%)) for both gamma cameras was low, and activity concentrations measured in images with Q.Metrix were consistent.

**2. Accuracy of measurements for the activity of hot spherical inserts in the NEMA/IEC NU2 Body Phantom.**

**(A)**

**Fig. 10:**
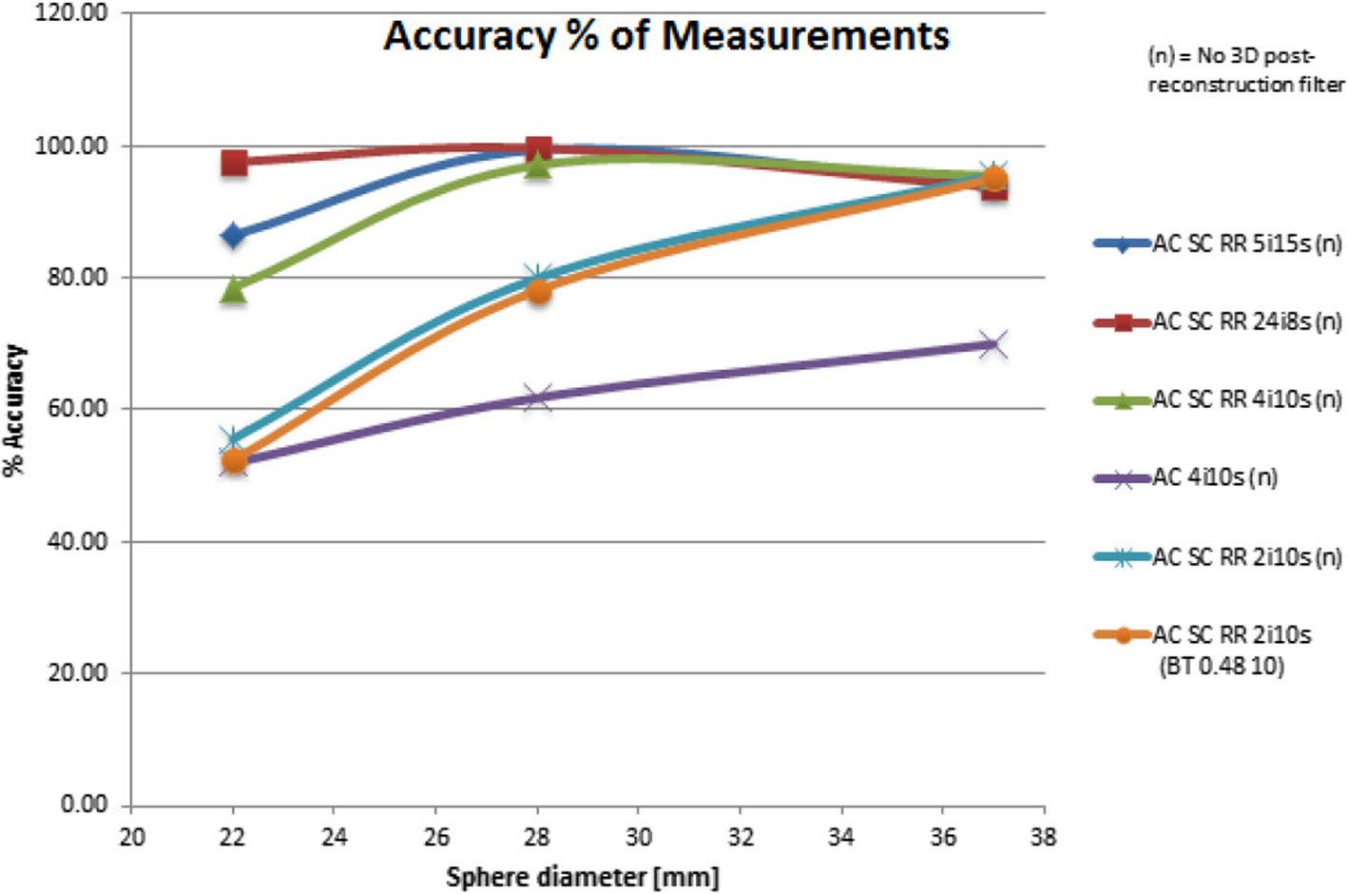
Accuracy of measurements for activity in the 3 largest spheres of the NEMA/IEC Body Phantom scanned in GE Healthcare laboratory. Source: White Paper, DOC1951185, 2017, GE [12]

The juxtaposition of Figs. 5 and 10 allows comparison of the accuracy of measurements for activity concentration obtained for the 3 largest spheres of the NEMA/IEC NU2 Body Phantom scanned in clinical Department 1 and in GE Healthcare laboratory. The images, acquired with different gamma cameras, were reconstructed using the same 6 methods.

The highest accuracy of measurements for the activity of hot spherical inserts was found, both in our study and in one performed by GE Healthcare [12], using an image reconstruction technique that involved 24 iterations, 8 subsets. For the two reconstruction techniques 5 iterations and 5 subsets or 4 iterations and 10 subsets, the accuracy of measurements for activity concentration in the largest sphere (ϕ 37 mm) was similar (approx. 90%). For two smaller spheres (ϕ 27 and 22 mm), the accuracy of measurement for activity concentration obtained in the clinical department of nuclear medicine was approx. 10% lower than that reported by GE Healthcare. The maximum value of the accuracy of the sphere activity measurement for 5 iterations in the GE Healthcare laboratory was as high as 99.4% for the 28-mm sphere. In our study, the accuracy of measurement for this sphere was 90.2%. GE Healthcare recognized 5 iterations and 15 subsets or 4 iterations and 10 subsets as the optimal reconstruction methods [12]. The results of deviations of activity concentration measurements from the expected values obtained in our study (Fig. 6) confirmed the clinical usefulness of the above reconstruction methods (averaged BIAS for all spheres ~ 40% with an average noise level not exceeding 4%). The algorithm with a large number of 24 iterations significantly extended the reconstruction time, increased the image noise and was considered a technique unsuitable for clinical applications.

**(B)**

The analysis of RC_max_ values obtained in the two departments for 3 methods of the NEMA/IEC NU2 Body Phantom reconstruction showed that the average of the differences of the corresponding recovery coefficients was within 10%, but the RC curves from Department 1 and Department 2 had different ranges of values (Fig. 8). Many factors could have contributed to the differences in the obtained results, including but not limited to: the method of preparing the phantom, the processing of the results by different operators using two different versions of the Xeleris software.

**Fig. 11:**
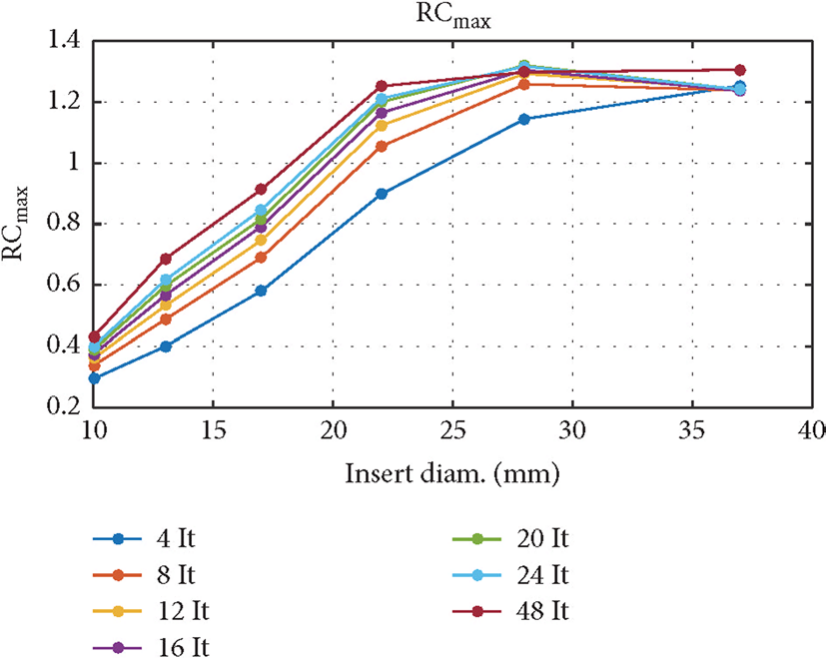
Recovery coefficients (RC_max_) for activity concentration in 6 spherical inserts of the NEMA/IEC NU2 Body Phantom scanned with Siemens Symbia Intevo SPECT/CT system [11] (Courtesy of Dr Silvano Gnesin)

In the study by Gnesin et al. [11], the acquisition of the NEMA/IEC NU2 Body Phantom was performed with Siemens Symbia Intevo camera. Acquired data were processed on Siemens Syngo workstation. Figure 11 presents recovery coefficients (RC_max_) for 6 spherical inserts reported by Gnesin et al. The colors of the RC_max_ curves assigned to individual reconstruction methods were consistent with the method markings in the legends of Figs. 7 and 8.

The general shape of the RC_max_ curves obtained in our work (Figs. 7, 8) was similar to those presented by Gnesin et al. [11] (Fig. 11). The authors of [11] used a different manufacturer's system (Siemens) and considered 16 iterations of 4 subsets to be the optimal method of reconstruction. Figure 9, obtained with the GE Healthcare camera, using analogous 7 reconstruction methods, does not clearly confirm the preference of the 16 iterations 4 subsets method. Reconstruction parameters influence activity recovery and thus SUV. It should therefore be emphasized that optimization is recommended for each clinical system, according to clinical requirements.

In the study from 2018, Collarino et al. [15] performed recovery coefficients measurements for six NEMA/IEC Body Phantom spheres using Discovery NM/CT 670 Pro GE Healthcare gamma camera. The shape of RC curves differed to some extent from the shape of analogous curves in our and Gnesin's work [11]. The authors of the work [15] emphasized the influence of many factors (including gamma camera sensitivity measurement techniques, reconstruction techniques, segmentation methods and count statistics in images) on the quantitative accuracy of SPECT/CT system. In the publication [1] from 2019, Peters and colleagues presented RC curves obtained using similar acquisition and reconstruction parameters to those used in our work and the work of Gnesin et al. [11] to analyze the NEMA/IEC Body Phantom. The shapes and values of the RC_max_ curves for Discovery NM/CT 670 Pro gamma camera were consistent with our results. A similar agreement was found for the results of Gnesin et al. [11] for the Symbia Intevo gamma camera. Peters et al. [1] compared five SPECT/CT systems from different vendors at different imaging centers for their quantitative capabilities for ^99m^Tc and found that: “the largest contribution for inter-system variation is due to vendor-specific reconstruction settings.” In the study from 2012, Seret et al. [16] compared four SPECT/CT systems and found that for objects which dimensions exceeded the SPECT spatial resolution by several times, quantification based on calibration procedure similar to the one used in PET was possible within a 10% error. In the study performed by Ryu et al. in 2019 [17], they confirmed that quantitative PET performance standards (NEMA NU 2-2012) apply to SPECT/CT imaging. In publications on the quantification of PET for ^18^F [7, 13], the authors reported (for the NEMA/IEC Body Phantom) deviations from Bg. cal = 1 less than 10%, COV less than or equal to 15%, and RC values remained stable and generally within ± 10% for the four largest and ± 20% for the two smallest spheres.

Data presented in Table 2 imply that it is possible to obtain repeatable results of the same tests and SPECT/CT acquisitions performed in two different medical departments with different gamma cameras. Values of hot contrast measured for the ϕ = 22 mm hot spherical insert in these departments corresponded to values reported by Gnesin et al. [11].

**3. Comparison of SUVs acquired for a single calibration source**

Data presented in Table 3 were used to calculate conversion factors for SUV in order to compare standardized uptake values obtained in the two medical departments. The objective comparison of SUVs is very important for monitoring the status of a patient being tested in different nuclear medicine departments with different SPECT/CT scanners.

Harmonization of SPECT/CT scanners for quantitative ^99m^Tc studies is feasible when proper scanner—activity meter cross-calibration and harmonized image reconstruction procedures are followed. In addition, if the activity measurements are performed with different activity meters, then the important step of full harmonization could be a cross-calibration of dose calibrators over the entire range of ^99m^Tc activities used in departments [18, 19].

Currently, medical facilities worldwide do not perform the clinical standardization of SUVs as a clinical routine. For this reason, doctors are unable to analyze changes in the standardized uptake value for imaged tumors if the patient has been examined with different scanners, even in the same nuclear medicine department. Clinical publications reporting SUVmax and SUVmean measured in patients’ images should provide SPECT/CT acquisition parameters, but also details regarding the image reconstruction technique [20–23]. This would allow for an initial comparison of quantification results for pathological changes in images acquired with different SPECT/CT systems.

For a correct interpretation of the clinical SPECT/CT image, it is advisable to know the reference values of the SUV for normal tissues. Currently published papers concern the possibility of identifying the stage of the disease as well as differentiating inflammatory and neoplastic lesions using the SUV index measured in clinical SPECT images [24, 25]. In this case, PET is a bit ahead of SPECT. Thanks to the standardization and harmonization of PET techniques, the absolute indicators of SUV PET can be used to plan radiotherapy, monitor treatment and also as a prognosticator—to predict overall patient survival [26]. In the study from 2021, Arvola et al. [27] showed that SUVs measured in SPECT images of breast and prostate cancer bone metastases were significantly correlated with SUVs obtained from PET images. Following the PET pattern, the basis for achieving absolute SPECT/CT quantification should be the harmonization of gamma cameras calibration procedures, acquisition parameters, image processing and analysis.

The EANM is currently working on a SPECT/CT harmonization pilot study. In the opinion of the authors of this work, the launch of the EARL program for quantitative standardization and harmonization of SPECT/CT would significantly increase the clinical importance of this diagnostic imaging technique.

## Conclusions

1. Measurements taken in our study confirmed the clinical suitability of 5 parameters of image quality (Bg. cal—background calibration factor, ∆*A*_tot_—total activity deviation, COV—coefficient of variation used for image noise assessment, *Q*_H_—hot contrast, AM—accuracy of measurements, or RC—recovery coefficient) for the validation of SPECT/CT system performance in terms of correct quantitative acquisitions of images.
2. This work shows that absolute SPECT/CT quantification is achievable in clinical nuclear medicine centers. Results variation of quantitative analyses between centers is mainly related to the use of different reconstruction methods.
3. It is necessary to standardize the technique of measuring the SUV conversion factor obtained with different SPECT/CT scanners.

## Data Availability

The datasets used and/or analyzed during the current study are available from the corresponding author on reasonable request.

## Abbreviations

SPECT/CT: Single-photon emission computed tomography/computed tomography
PET: Positron emission tomography
GE: General Electric
^99m^Tc: Technetium-99m
NM: Nuclear medicine
EANM: The European Association of Nuclear Medicine
EARL: EANM Research Ltd
QIBA: Quantitative Imaging Biomarker Alliance
Euratom: European Atomic Energy Community
MBq: MegaBecquerel
POLATOM: Part of the National Centre for Nuclear Research in Poland
keV: Kiloelectron volt
AC: Attenuation correction
SC: Scatter correction
RR: Resolution recovery
Bg. cal: Background calibration factor
∆*A*_tot_: Total activity deviation
COV: Noise level estimation
NEMA/IEC: The National Electrical Manufacturers/The Association International Electrotechnical Commission
VOI: Volume of interest
*Q*_H_: Hot contrast
AM: Accuracy of measurements
FWHM: Full width at half maximum
RC: Recovery coefficient
ROI: Region of interest
SUV: Standardized uptake value
SUVmax: Maximum standardized uptake value
SUVmean: Mean standardized uptake value
